# Macrocystic lymphatic malformation with an unusual late presentation in a seven-year-old child: a case report and literature review

**DOI:** 10.1093/jscr/rjag490

**Published:** 2026-06-19

**Authors:** Mashael S Mahnashi, Abdulrahman A Otaif, Bashayr N Alsuwayt, Manal Bin Manie

**Affiliations:** Department of Otolaryngology–Head and Neck Surgery, King Abdulaziz University Hospital, King Saud University, PO Box 245, Riyadh 11411, Riyadh Province, Saudi Arabia; Faculty of Medicine, Jazan University, AlMaarefah Rd., Jazan 45142, Saudi Arabia; College of Medicine, University of Hail, Hail 81422, Hail Region, Saudi Arabia; Department of Otolaryngology–Head and Neck Surgery, King Abdulaziz University Hospital, King Saud University, PO Box 245, Riyadh 11411, Riyadh Province, Saudi Arabia

**Keywords:** lymphatic malformation, lymphangioma, delayed presentation, pediatric neck mass, macrocystic, surgical excision

## Abstract

Lymphatic malformations are rare congenital lesions usually presenting at birth or early childhood, with delayed cases in school-age children being uncommon. We report a 7-year-old girl with a 9-month, painless, enlarging left neck mass without infection or trauma. Exam showed a soft, compressible mass in the posterior cervical triangle. Magnetic resonance imaging revealed a multiloculated cystic lesion consistent with a macrocystic lymphatic malformation. Surgical excision confirmed lymphangioma, and the recovery was smooth. This case underlines the rare delayed presentation of a congenital lesion and the importance of including lymphatic malformations in pediatric neck mass diagnosis, regardless of age, highlighting imaging, and surgery’s role in good outcomes.

## Introduction

Lymphatic malformations, also known as lymphangiomas, are rare benign congenital lesions resulting from abnormal embryological development of the lymphatic system that predominantly affect the head and neck region [1]. Most lymphangiomas present at birth or in early infancy, with 67% diagnosed before the age of 2 years [2]. Delayed presentation in school-age children is uncommon and represents an unusual manifestation of these congenital lesions.

## Case report

A 7-year-old girl, born full-term via normal vaginal delivery with no significant past medical or surgical history, presented to the Ear, Nose, and Throat (ENT) clinic with a left-sided neck mass that had appeared suddenly nine months prior to presentation and progressively increased in size. The swelling was painless and was not associated with preceding infection, fever, or rapid enlargement during upper respiratory tract infections. There was no increase in size with coughing or straining. The patient denied respiratory distress, voice changes, sleep apnea, or speech difficulties. There was no relevant family history.

On examination, the patient was vitally stable. ENT examination revealed a clear throat and bilaterally intact external auditory canals and tympanic membranes. Flexible nasal endoscopy demonstrated patent nasal cavities bilaterally, mild inferior turbinate hypertrophy, ~50% adenoid hypertrophy, and bilaterally mobile vocal cords with symmetric movement.

Head and neck examination revealed a large left-sided neck mass located mainly in the posterior cervical triangle, measuring ~4 × 5 cm. The mass was lobulated with a smooth surface, soft in consistency, fluctuant and compressible, with normal overlying skin. It was non-tender, without visible pulsations, and did not move with swallowing. The right side of the neck was normal with no palpable lymphadenopathy.

Magnetic resonance imaging (MRI) of the neck demonstrated a multiloculated, septated cystic lesion centered in the left posterolateral lower neck extending into the supraclavicular region, measuring ~52 × 45 × 40 mm. The lesion showed high T2 signal intensity with peripheral and septal enhancement and no solid component, without invasion of surrounding cervical musculature or vascular encasement. No cervical lymphadenopathy was identified. These findings were suggestive of a macrocystic lymphatic malformation (Figs 1 and 2).

**Figure 1:**
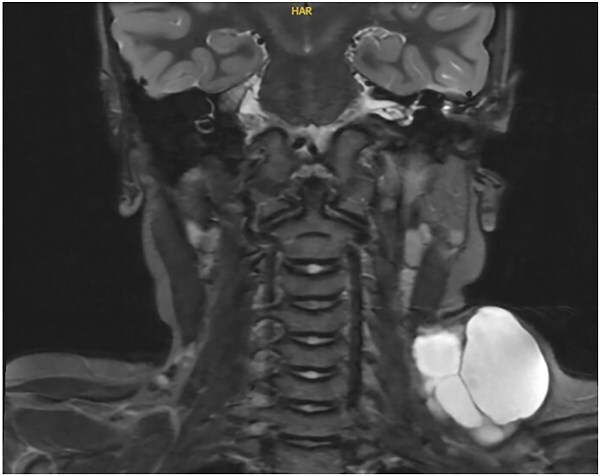
Coronal T2-weighted MRI of the neck demonstrating a well-defined multiloculated hyperintense cystic lesion in the left posterolateral lower neck extending into the supraclavicular region.

**Figure 2:**
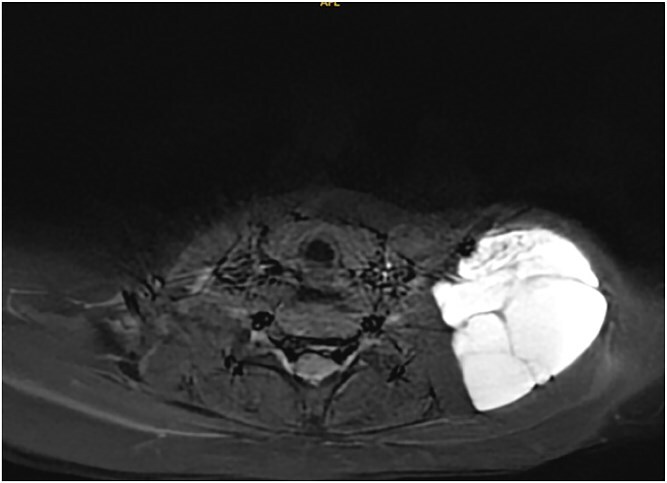
Axial T2-weighted MRI showing a large hyperintense multilobulated cystic lesion in the left posterior cervical triangle causing displacement of adjacent soft tissues without evidence of invasion.

The patient subsequently underwent complete surgical excision of the lesion. Intraoperatively, a multiloculated cystic mass was identified within the left posterior cervical triangle and carefully dissected and removed (Fig. 3). The excised specimen consisted of a multilobulated cystic mass, consistent with the radiologic findings (Fig. 4).

**Figure 3:**
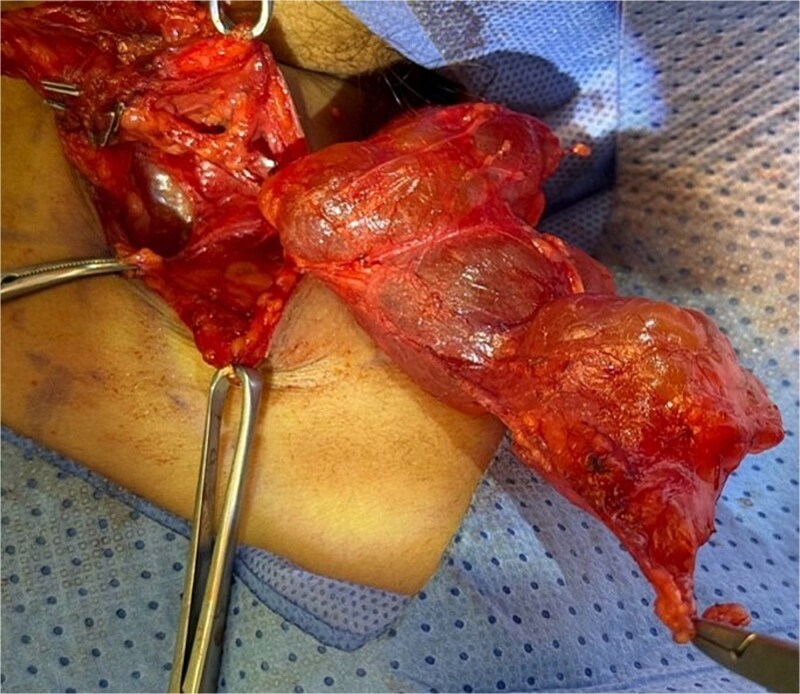
Intraoperative image demonstrating surgical excision of a multiloculated cystic lesion from the left posterior cervical triangle consistent with macrocystic lymphatic malformation.

**Figure 4:**
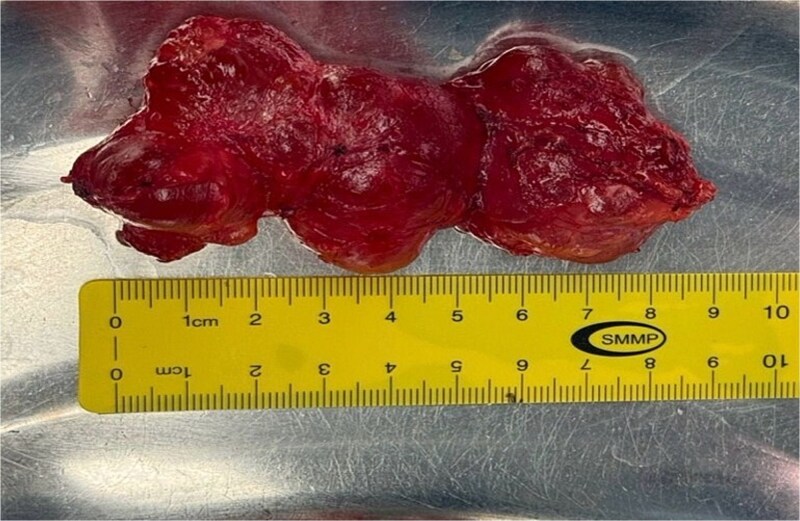
Gross specimen after surgical excision showing a multilobulated cystic mass.

Histopathological examination confirmed the diagnosis of lymphangioma (macrocystic lymphatic malformation), demonstrating dilated lymphatic vascular spaces. Immunohistochemistry showed positivity for D2–40 and CD31 and negativity for Pan-CK, supporting a lymphatic endothelial origin.

The postoperative course was uneventful, and the patient was discharged in stable condition with no immediate complications.

## Discussion

This case presents a remarkable delayed presentation of macrocystic lymphatic malformation in a 7-year-old girl, representing a significant deviation from well-established epidemiological patterns in the literature. The timing of presentation is particularly striking when compared to large series data. Eliasson *et al.* documented that 67% of lymphatic malformations are diagnosed before the age of 2 years in their comprehensive 8-year population study from a national treatment center [2]. This finding is strongly supported by Chen *et al.*, who reported that 72.4% of giant macrocystic cervicofacial cases presented before age 3, with only 5 of 47 patients reaching school age before diagnosis [3]. Na *et al.* provided additional evidence of early presentation patterns, noting that 83% of patients were treated before age 7 [4]. Our patient’s presentation at exactly age 7 therefore places her at the extreme end of the typical presentation spectrum.

The spontaneous appearance of the lesion nine months prior to presentation, occurring without identifiable precipitating factors, adds considerable clinical interest to this case. Rahbar *et al.* specifically investigated delayed presentations and documented a similar case in a 7-year-old boy, emphasizing that such late manifestations are typically triggered by trauma or infection rather than spontaneous development [5]. This observation is corroborated by Phang *et al.*, who studied spontaneously resolved macrocystic lymphatic malformations and found that changes in lesion behavior were commonly associated with local or upper respiratory tract infections [6]. The complete absence of such triggers in our patient makes this presentation exceptionally unusual and challenges conventional understanding of delayed manifestation mechanisms.

Review of similar cases in the literature reveals varying patterns of delayed presentation across different anatomical locations and age groups. Ozdemir *et al.* reported a particularly instructive case of retroperitoneal lymphangioma with initial presentation at age 7, which subsequently recurred 11 years later, highlighting important long-term considerations for delayed presentations [7]. Hurwitz *et al.* documented scrotal lymphangiomas with mean presentation age of 3 years, noting universal preoperative misdiagnosis, which underscores the diagnostic challenges inherent in delayed presentations [8]. The literature also documents even more extreme delayed presentations extending into adulthood, with Naidu *et al.* providing a comprehensive review of 91 adult cases reported since 1828, and Colangeli *et al.* describing a 56-year-old patient, demonstrating that lymphatic malformations can remain clinically dormant for decades [9, 10].

The anatomical location in the left posterior cervical triangle observed in our patient aligns well with established distribution patterns. Na *et al*. identified the posterior triangle as the most common cervical location, accounting for 54% of cases, while Cho *et al.* confirmed frequent neck involvement in their retrospective review of cervicofacial presentations [4, 11]. The macrocystic morphology, characterized by multiloculated septated appearance on MRI with high T2 signal intensity, corresponds precisely to imaging patterns described by Diarra *et al.*, who emphasized MRI’s crucial role in both diagnosis and surgical planning [12].

The successful complete surgical excision achieved in this case reflects the generally favorable outcomes associated with macrocystic lesions in older pediatric patients. Hassan *et al.* reported complete surgical excision in 88.9% of giant macrocystic cases without significant neurovascular injury, with satisfactory cosmetic results in 88.9% of patients [13]. Chen *et al.* achieved surgical radicality in 89.4% of cervicofacial cases, though importantly noted higher recurrence rates in newborns compared to older children, suggesting that delayed presentation may actually confer certain surgical advantages [3].

This case contributes meaningfully to the limited literature on spontaneous delayed presentations of lymphatic malformations, emphasizing the importance of maintaining these lesions in differential diagnosis considerations regardless of patient age or absence of typical precipitating factors.
